# Acceptability of intravenous propofol sedation for adolescent dental care

**DOI:** 10.1007/s40368-019-00482-0

**Published:** 2019-10-08

**Authors:** C. Dixon, A. Aspinall, S. Rolfe, C. Stevens

**Affiliations:** 1 University of Manchester, Manchester, UK; 2 Birmingham Dental Hospital, Birmingham, UK; 3 Manchester University Foundation Trust, Manchester, UK; 4 Manchester University NHS Foundation Trust, University of Manchester, Manchester, UK

**Keywords:** Propofol, Intravenous sedation, Adolescent, Dental anxiety, Target-controlled infusion

## Abstract

**Purpose:**

Propofol is an intravenous anaesthetic agent commonly utilised in general anaesthesia, however in sub-anaesthetic concentrations can be utilised to provide sedation through automated dosing of target-controlled infusion (TCI). TCI has been shown to provide accurate and stable predicted plasma and effect-site concentrations of propofol. A four-part mixed-method prospective study was undertaken to evaluate the safety and patient acceptability of intravenous propofol sedation in adolescent patients requiring dental care. There is a paucity in the literature on patient-reported outcomes and patient safety in the management of adolescent patients for dental treatment.

**Methods:**

Demographics were recorded including age, gender, ASA Classification and Children’s Fear Survey Schedule—Dental Subscale (CFSS-DS) completed pre-operatively. Behaviour ratings of the Frankl and Houpt scales were recorded followed by post-operative questionnaire and telephone consultation. Consultation was completed following the procedure to determine patient satisfaction, memory of the procedure and any reported side effects of treatment. Qualitative thematic analysis was utilised.

**Results:**

55 patients were recruited for the study, of which 49 (mean age 14.67 years) completed the sedation study and were treated safely with no post-operative complications. The mean lowest oxygen saturation was 98.12% SpO_2_ (SD 2.6). Thematic analysis demonstrated positive patient-reported outcomes to IV sedation.

**Conclusion:**

Propofol TCI sedation is an effective treatment modality for the management of dentally anxious adolescents as a safe alternative to general anaesthesia, allowing the opportunity for increased provision of treatment per visit on those patients with a high dental need. Further randomised controlled trials comparing propofol TCI to other pharmacological managements are required.

## Introduction

For the majority of anxious children, dental treatment can be provided using careful behavioural management, with some requiring the adjunct of conscious sedation to complete treatment. Inhalation sedation (IHS) with nitrous oxide and oxygen is the preferred technique for initial management for those who require conscious sedation under the Scottish Dental Clinical Effectiveness Program Sedation Guidelines (SDCEP 2017), with literature supporting this successful, safe and well-tolerated treatment modality from children aged 4 years (IACSD 2015; NICE 2010; EAPD 2003). The success of IHS is supported by a foundation of core behavioural management techniques, appropriate titration of nitrous oxide to the safest effective concentration allowing patients to accept dental treatment and enabling cooperation over multiple visits (Major et al. 1981).

However, in the adolescent population, success is sometimes compromised where patients present with severe dental anxiety and/or complex dental treatment (Shaw and Niven 1996) and these patients may require alternative sedation or general anaesthesia. For those adolescent patients who are anxious, it is preferable to carefully select the most appropriate pharmacological technique for the individual patient to avoid progressing through a range of techniques that may possibly fail based on their clinical and dental need (SDCEP 2017).

The Department of Child Dental Health, University Dental Hospital of Manchester, established a regional referral service for anxious adolescent children requiring dental treatment in 2009. The adolescent intravenous sedation (IV) service was established to provide an alternative treatment provision to general anaesthesia for anxious adolescent patients requiring restorative and/or oral surgery care.

Propofol 2,6-disopropylphenol is an IV anaesthetic agent formulated as an emulsion in 10% soybean oil, 2.25% glycerol, 1.2% purified egg phosphatide and disodium edetate (EDTA) which is commonly utilised in general anaesthesia as a bolus induction agent. It results in rapid loss of consciousness and loss of airway tone, with cardiovascular and respiratory depression (Hosey et al. 2004). As such, the administration of propofol can only be provided by a qualified anaesthetist and is described as an advanced technique (SDCEP 2017). Side effects patients often report are discomfort and pain on administration of IV propofol, which has been mitigated in some studies with IV administration of 1 ml of lidocaine (Tan and Onsiong 1998).

Propofol is an isotonic formula with a neutral pH and is extensively bound to the plasma proteins (95–98%), and therefore does not trigger a histamine release to those with soya or egg allergy (Irwin et al. 1997). The pharmacokinetic properties of propofol are very advantageous with an initial distribution half-life of 2–8 min and terminal elimination from 4 to 24 h dependent upon the infusion dose (Oei-Lim et al. 1998). The hypnotic potency has been recorded to cross the blood–brain barrier ranging from 1.5 to 2.9 min with rapid distribution into peripheral tissues (Oei-Lim et al. 1998).

The pharmacokinetic properties of propofol also allow its utilisation as a sub-anaesthetic agent to provide sedation through target-controlled infusion (TCI), enabling sedation to be tailored to the individual patient (Lee 2004). TCI maintains a constant sedation level by utilising a computerised propofol dosing regimen, combining a real-time pharmacokinetic model with an infusion pump and has several algorithm settings. This enables the administration of propofol to be maintained as a selected constant blood concentration. Allowing rapid alterations to be made when additional sedation is required, such as for local anaesthetic administration or surgical treatment (Milne and Kenny 1998) in comparison to bolus administration, which can provide varying effects with peak and trough concentrations of propofol (Lee 2004).

TCI pumps have also been further expanded to incorporate a handset “patient maintained pump”, enabling patients to titrate the sedative effect to their own requirements, with the patient-controlled feedback loop (Irwin et al. 1997). TCI patient-controlled pumps have been shown to be effective in the reduction of pre-operative anxiety prior to surgery, with no reported over sedation or cardiovascular instability in endoscopic examinations (Milne and Kenny 1998).

Propofol has an effect on explicit memory at any dose, producing a hypnotic effect by a positive modulation of the inhibitory function of the neurotransmitter gamma-amino butyric acid (GABA) receptors. This results in stimulation in the inhibitory system and provides sedation with a generalised global depression of the synaptic activity in the brain (Oei-Lim et al. 1998). Propofol’s amnesic and anxiolytic effects have been demonstrated as aids for clinical outcomes in the management of anxious patients and or complex dental treatment (Hosey et al. 2004).

The aim of this study was to evaluate safety and patient acceptability of IV propofol sedation in adolescent patients requiring dental care at the University Dental Hospital of Manchester.

The objectives were:

- To determine the percentage of patients who are safely treated with IV propofol.
- To report on the postoperative satisfaction of patients who receive propofol sedation.
- To pilot data collection methods to record patient, operator and anaesthetist acceptability of IV propofol.
- To facilitate sample size calculation for future research development.

## Materials and methods

**Fig. 1:**
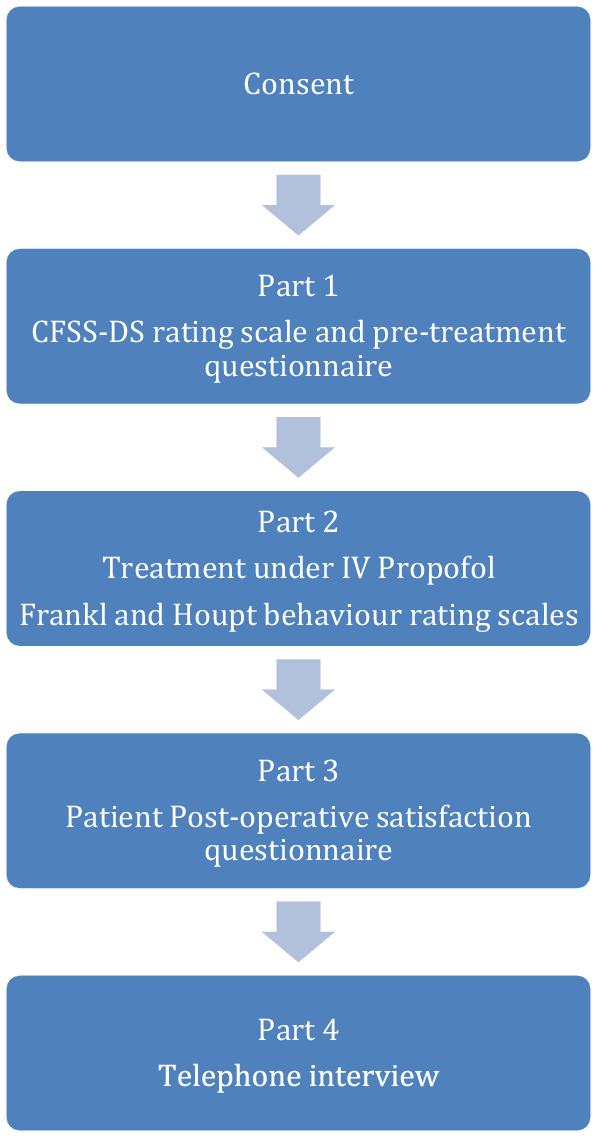
Flowchart of mixed-method IV propofol study

The study was completed at the University Dental Hospital of Manchester in four phases; a pre-treatment questionnaire, routine monitoring during treatment, recording of patient’s ability to accept treatment by the operating dentist and anaesthetist. The final section included a post-treatment questionnaire with data collection via telephone consultation (Fig. 1). Ethical approval by NHS Research Ethic Committee Reference 10/H1016/81. Informed consent was obtained from all individual participants included in the study. A sample size calculation was undertaken to set a confidence interval of 3% in either side with the sample size of 43.

### Inclusion criteria

- The child has the intellectual or emotional maturity to cooperate with the planned procedure (minimum age of 12 years old).
- This child is anxious but willing to attempt treatment with IVS.
- The patient is under the age of 16 years at time of consent and eligible for treatment in the paediatric setting.

### Exclusion criteria

- The child is unwilling to attempt treatment whilst awake.
- The child is not ASA I or II and would be more appropriately managed in a children’s hospital setting.
- The child does not comprehend the English Language.

### Analysis of data

The data collection was anonymised and recorded on Microsoft Excell 2010 with statistical analysis completed on SPSS V24. A significance level of *p* = < 0.05 was agreed. Frankl scores from operator and anaesthetist were assessed for agreement using a Cohen Kappa analysis. All telephone calls were undertaken by the same dental clinicians (AA and CS), and were transcribed verbatim and data analysis was completed using thematic analysis (Braun and Clarke 2006).

## Results

**Fig. 2:**
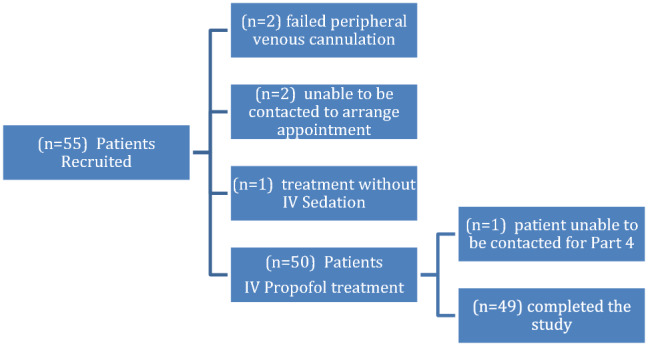
Flowchart of study recruitment and participation

A total of 55 patients were recruited and 49 successfully completed treatment under the IV propofol sedation and four-part study (Fig. 2). The majority of patients who required treatment were female (63%), with the mean age being 14.67 years (12.6–16.8 range). Of those recruited the majority of patients were healthy ASA 1 classification, with asthma the most common comorbidity reported (20%) (Table 1).

**Table 1:** Patient demographics recruited in the study

Patient demographics	
Gender	Female: *n* = 31 Male: *n* = 18
Age	14.67 years mean 12.6–16.8 years range
ASA classification	1: 67% (*n* = 33) 2: 33% (*n* = 16)
Medical history	Asthma (*n* = 10) Eczema (*n* = 6) ADHD (*n* = 3) Type 1 diabetes (*n* = 1) Migraines (*n* = 1) High BMI (*n* = 2)

**Fig. 3:**
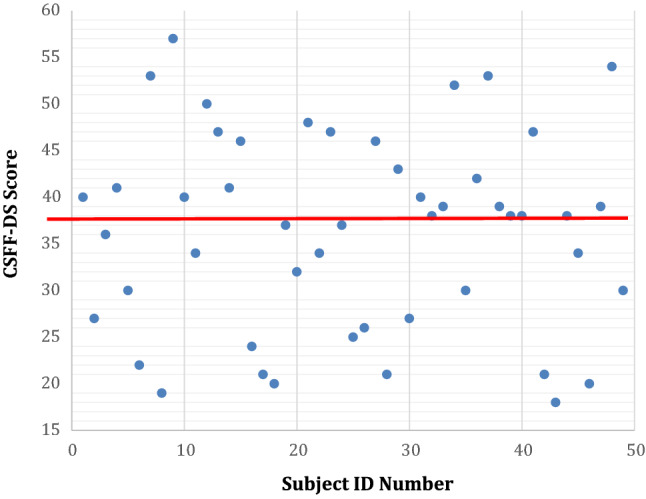
CSFF-DS results of patients’ IDs, red line indicated a score of 38

The pre-operative CFSS-DS (Fig. 3) recorded a mean response score of 36.3 (sd 10.6) with a range of 18–57. Those scores recorded above 38 (red line) have been clinically shown to be indicative of dental fear (Cuthbert and Melamed 1982). Preoperative anxiety levels on average was higher in females 37.5 (sd 11) than males 34.3 (sd 9.7), however there was no statistical significance difference between CFSS-DS and gender. The most common questions to elicit a high level of response were “injections” (4.1 ± 0.65) and the “sight of the dentist drilling” (3.5 ± 0.72).

**Fig. 4:**
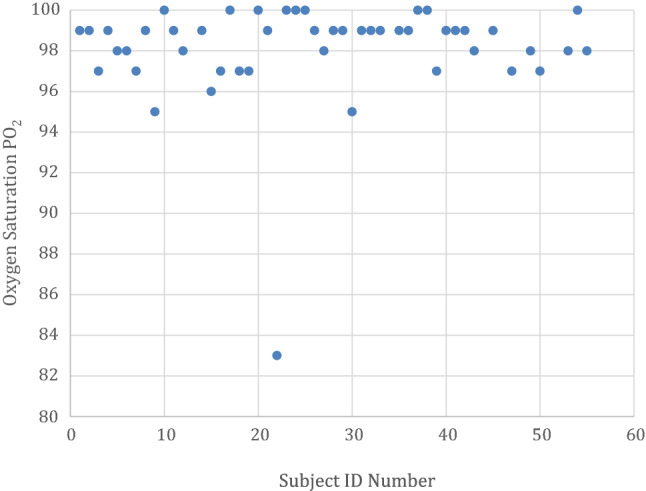
Lowest oxygen saturation PO_2_ recording per patient

Peripheral oxygen saturation (SpO_2_) was recorded in all cases, with one patient reported to hyperventilate during the placement of a dental filling and clenching of fists was attributed to the SpO_2_ drop in oxygen saturation to 83% SpO_2_, the patient was subsequently calmed down, and within 30 s, the patients SpO_2_ levels had returned to 100% SpO_2_. The clinical notes of the patient note apart from this incident the patient was communicating well throughout and reported an uneventful recovery. All other patients recorded peripheral oxygen saturation within the desired window of safety throughout the procedures with an average recording 98.12% SpO_2_ (sd 2.6) (Fig. 4). Throughout treatment base line monitoring of ECG, blood pressure at 5-min intervals and oxygen saturation in line with departmental sedation protocol.

The operating time of procedures varied as expected due to the variety of treatment provided with average recorded treatment of 31.8 min (sd 13.5) (Table 2). The average received titrated total dose 211.18 mg of propofol (96.7–366 mg range). The majority of patients were recorded as Frankl Score “definitely positive” by both dental and anaesthetic clinician with a Cohen Kappa analysis of the Frankl Score (*k* = 0.931) demonstrating good inter-rater reliability within the data set. The overall Houpt Score demonstrated a score of “excellent, no crying or movement” in the majority of patients (74%). Only two patients recorded a score of three where treatment was interrupted due to cooperation, but treatment was eventually completed with no complications.

**Table 2:** Baseline data collection recordings of patients

Data collection		
Dental treatment provided	Extraction 59.2% (*n* = 29) Surgical extraction 4.0% (*n* = 2) Restorations 8.2% (*n* = 4) Extractions and restorations 28.6% (*n* = 14)	
Operating time	8–63 min range (sd 13.5) 31.8 min average	
Dentist Frankl Score	4	41 (84%)
	3	6 (12%)
	2	2 (4%)
	1	0 (0%)
Anaesthetist Frankl Score	4	40 (82%)
	3	7 (14)
	2	2 (4%)
	1	0 (0%)
Overall Houpt Behavioural Score	6	36 (73%)
	5	6 (12%)
	4	5 (10%)
	3	2 (4%)
	2	0 (0%)
	1	0 (0%)
Recovery time	35 min average (sd 9.2) 60–20 min range	

In all cases recovery was uneventful, with patients reported to be alert and able to safely walk unaided prior to leaving the sedation suite. Patient one’s recovery was initially set at 1 h, which on clinical reflection was noted as unnecessary and further patient recovery times were reduced accordingly (Table 2). The majority of treatment required for the cohort under IV sedation was recorded as simple exodontia (59.2%), followed by quadrant dentistry including extractions and restorative care (28.6%). The type of treatment provided was not statistically significant on pre-operative CFSS-DS score (*p* = 0.822).

Following appropriate recovery and prior to discharge, the patients were provided with a post-operative questionnaire with a Likert Scale of 5 strongly agree to 1 strongly disagree. Quantitative feedback from the patients was very positive 96% strongly agreed that the dental team were “friendly and approachable” and 90% of patients strongly agreed they would attend for the same dental procedure again. There were no patients who reported that they would definitely not have the procedure again, or were not likely to recommend the service to friends and family. It was recorded that 94% of patients found the effects of sedation enabled them to feel “more relaxed” and subsequently able accept dental treatment.

Data analysis was completed using thematic analysis (Braun and Clarke 2006) for operative and telephone consultation arms of the study. Thematic analysis enables flexibility in its methods for identification of themes to further analyse and report on clear detailed patterns found within the data set. Braun and Clarke (2006) describe a six-phase guide, which has been utilised for this data.

A pattern on initial anxiety prior to and during venous cannulation was noted, however once IV propofol infusion had begun, the majority of patients were cooperative and relaxed with verbal communication maintained throughout as reported by the anaesthetist. “Very anxious during cannulation, tearful, once sedation in progress very cooperative” Patient 13. “Good, silent tears after cannulation, but relaxed, communicating throughout” Patient 43. This can be attributed to the positive pharmacological effects of propofol with rapid onset to achieve desired sedation (Tan and Onsiong 1998). Barriers, which may have increased anxiety of patients and cooperation were noted in difficulty of venous access: “Difficult venous access—hand in warm water, excellent sedation and cooperative” Patient 43. “Very anxious, screaming at cannulation, very challenging more settled with sedation but intermittently very upset, verbal communication throughout, treatment carried out but difficult” Patient 43. It has been shown that fear and anxiety can activate the sympathetic nervous system, which may result in vasoconstriction of the peripheral veins, increasing the difficulty in cannulation. These repeat attempts provide further distress for the patient and require a skilled and experienced operator to manage the patient (Lenhardt et al. 2002). In anxious patients, warming of the hands in warm water to encourage peripheral dilation was provided on two occasions to aid cannulation. Other patients were less cooperative requiring coaxing and positive reinforcement to enable successful cannulation. Two patients refused cannulation, which highlights a barrier if the management of those patients who refuse cannulation with a significant dental/needle anxiety and if additional services such a cognitive behaviour therapy (CBT) would need to be explored.

The dental clinician’s comments note a strong theme within the data set of good cooperation with patients communicating throughout treatment under IV propofol. On several occasions, it was noted that patients asked for further treatment to be completed on the same visit. In those that found treatment difficult, this was usually attributable to invasive procedures such as local anaesthetic administration, or the anticipation of the injection. Clinical notes reported an increase in treatment time, and requirement of increasing TCI infusion rate to achieve the desired sedative effect. This highlights the need for good behavioural management skills from all the clinical team tailored to each individual patient to enable successful treatment outcomes. “Movement with local anaesthetic. Needed coaxing. Excellent with extractions maintaining verbal contact throughout” Patient 38 and “Co-operative throughout, became teary after realising local anaesthetic had been administered” Patient 41.

### Telephone questionnaire

**Fig. 5:**
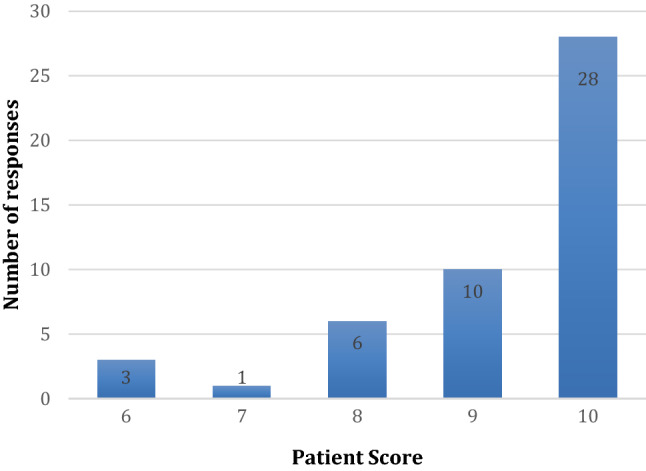
Patient-reported feedback on level of sedation (1 = poor to 10 = excellent)

A structured telephone questionnaire was completed with the patients the day following treatment. The interviews were aimed at exploring patient-reported outcomes following IV propofol sedation. The majority of patients commented they found sedation to be pleasant (89.8%) with a Likert Scale of 1 = poor to 10 = excellent on the level of sedation presented in Fig. 5.

### Propofol effect on memory

The amnesic effect of propofol has been widely reported and explored post-operatively, in particular looking at recall of patients’ treatment. 91.5% (*n* = 43) of patients had no memory of their procedure or local anaesthetic administration which was statistically significant *p* = <0.0011. All subjects were shown an A4 image of a red mini-car prior to treatment for 10 s, on asking the colour of the object only 14.5% (*n* = 7) of the subjects could recall the colour of the car.

On further discussion of any memory of the dental procedure, the cannulation “needle in hand” was the most common memory patients reported. Several patients reported no memory of the entire procedure or reported the event felt like a blur, which is attributed to the amnesia effect of propofol. “That I kind of fell asleep, do not remember anything and do not feel anything” Patient 39 “did not remember much, helped cope with dental treatment” Patient 29 “the fun of it”, “nurses were really nice” I thought it was mad that I could not remember anything “Mum told me I shouted out I love Spain but I cannot remember it” Patient 40.

An awareness of the treatment was also discussed by several patients, with memory of people talking, and music playing but no clear recollection of the event or treatment. In those that reported memory of the treatment procedure, it was described in a positive light; “remember stitches, I remember the medicine made me feel nice and it was a good feeling” Patient 16, “kind of remember tooth coming out but did not feel anything” Patient 32.

“Remember feeling tooth coming could, could cope with it, the sedation helped” Patient 9.

### Post-operative side effects

The study also reviewed the post-operative experiences of the IV sedation of the patients when they initially left the hospital and the next day. The majority of patients reported no initial sedative side effects following discharge from the hospital with 40.8% of patients reporting side effects the following day. Drowsiness was the most common side effect following sedation (*n* = 6), and pain at cannulation site (*n* = 6) and one patient reporting bruising in the back of the hand where the cannula was placed. Headaches were reported from three patients, with one patient reporting nausea the following morning. Our standard clinical practice required all patients have the following day off school and to be cared for by a responsible adult.

Patient-reported effects of IV sedation were explored with the anxiolytic effect of propofol sedation being a common theme, as the patients felt a strong sense of relaxation during the treatment. This was also commented on by the dental and anaesthetic team and was important in producing positive treatment outcomes. Some patients expressed that they felt without the IV sedation they would not be able to cooperate for the dental treatment, and this outcome was reflected positively. “I was calm not worried” Patient 3. “Very relaxed felt half asleep” “everything felt blurred” “would not have had injection/extraction otherwise” Patient 7. “That at every point I was reassured by everybody it would be fine. Do not think would have coped without sedation would have been a lot more worried” Patient 19. A positive reported outcome was the effect on memory, with patients pleased they could not remember difficult or anxiety-provoking treatment such as local anaesthetic or having a dental extraction.

It was highlighted that patients were satisfied with the treatment provided with several noting an improvement in their oral health, including the management of symptomatic teeth with a reported improvement in their oral health. “Can eat properly now as had tooth out” Patient 34. An important element highlighted was the empathy of the dental team, which was recalled by patients in their experience of the sedation, reporting the holistic approach to their treatment enabled them to overcome difficult procedures such as cannulation or dental extractions. “Do not remember most of it…was quite worried the cream really helped the staff were very supportive” Patient 50; “not remembering it” “liked how friendly everyone was” Patient 43.

Throughout the qualitative feedback the negative memory of venous cannulation was noted by patients. Though all patients received Ametop Gel^®^ topical anaesthetic to the dorsum of the hand, patients still reported negative memory attributed to cannulation. The sensation of propofol was also reported as uncomfortable or painful by several patients. This is in line with current systematic reviews that note the lipid-based emulsion has been reported to cause pain in patients from between 28 and 90% of patients (Picard and Tramèr 2000). “Did not like tingling feeling as medicine went up my arm” Patient 42. “Feeling it going up my arm, felt cold, tingly and heavy. Not really bad but if have to pick one thing that was bad this would be it!” Patient 37. “Needle in back of hand at beginning. I felt it and I did not like it” Patient 40. “Stinging up arm” and “needle” Patient 26.

The post-operative effects of dental treatment following local anaesthetic were reported as a negative side effect. Patient 43 had attended for orthodontic extractions of four premolars, which attributed to the response of numbness following sedation in all quadrants. The post-operative anaesthesia is difficult to mitigate due to the nature of the oral surgery procedures and requirement for clinical care. “Feeling of numbness after sedation” Patient 5.

A negative response to a perceived loss of control was noted by some patients, with embarrassment at being unable to remember conversations during the procedure, and confusion following procedure regarding the treatment that had been provided. “Embarrassed about not remembering what I said” Patient 7. “Afterwards when I came round I was not sure if it had been done, finished and felt a bit confused” Patient 19.

An important theme to discuss is that several patients had no negative thoughts on their sedation experience, reporting it in a positive outcome. “Nothing it was good” Patient 15.

Feedback from patients on their overall thoughts of the IV sedation was discussed to review any suggested improvements for the service provision. Though many felt very positive about the service and treatment provision, those with suggestions requested more information about the exact treatment that would be happening on the 1st appointment, to potentially aid preoperative anxiety and a reduction in the waiting time prior to treatment. “The time waiting was the worst as I got worked up, If could have something to take mind off it and wait less would be better” Patient 8. “Improvements could be to have more information beforehand about the treatment, see the team and rooms would help make feel less nervous”. Patient 37 “Really good I do not think anything could be made better” Patient 5.

## Discussion

This study aimed to investigate the acceptability of IV propofol sedation for adolescent dental care, and patient-reported outcomes. Patient safety was recorded in all cases with no significant complications, and safety outcomes support previous literature by Hosey et al. (2004) and Alexopoulos et al. (2007). The use of a TCI technique enabled the anaesthetist to tailor the infusion rate of propofol dependent to the treatment complexity and anxiety of the patient throughout the treatment, with patients reported to be alert and fit for discharge around half an hour following treatment.

The majority of patients consented for the study were female, which supports previous literature that female adolescents are more anxious about dental treatment or more likely to report it and request treatment under sedation modalities (Hosey et al. 2004). Interestingly those patients who reported the highest CFSS-DS in this study were male, though this was not statistically significant and it would require a significantly larger cohort to evaluate this. In the recognition memory test, the study demonstrated impairment in the storage of new information and a significant propofol effect on ‘memory retrieval’ of the event. Alexopoulous et al. (2007) in their prospective study of patient’s pre- and post-operatively demonstrated no significant difference in reported anxiety scores. The amnesic effect of propofol sedation could be thought of as a positive effect, in that the patients do not remember treatment that they may have found difficult. Conversely, those patients who demonstrated excellent cooperation may be unable to remember their own success, reducing the potential to acclimatise to future dental treatment. There is also the risk that these patients may become reliant on sedation for dental care, which is a very resource-limited service in the NHS Dental Service for adult patients. In order to fully evaluate how propofol impairs memory, further extensive memory function tests including behavioural testing are required along with qualitative research to evaluate patient’s experience of IV sedation and how it impacts on their reported dental anxiety following treatment.

The study demonstrated a high acceptance rate for cooperation with patients first experience of propofol requiring no acclimatisation appointments to enable treatment. The reported side effects from treatment were noted as pain at the site of cannulation, and itching of nose through treatment (possibly due to nasal cannulae delivering oxygen). In all patients who cooperated enough to allow cannulation, treatment was successfully completed. All patients who attended for IV treatment were provided with Ametop Gel^®^ on the dorsum of the hand and behavioural management techniques were employed, however this did not mitigate the reported outcomes. The provision of nitrous oxide inhalation sedation and coolant spray could be explored as methods to reduce pain. However, previous studies by Paul et al. demonstrated no statistical difference between Ametop and nitrous oxide in the reduction of patient-reported outcomes (Paut et al. 2001). Minimising cannulation pain or discomfort is a challenge, and since at present there is no clinical alternative to enable IV treatment it is vital to manage patients’ expectations.

IV propofol sedation provides a safe and well accepted treatment option for anxious adolescent patients including those who have a history of failed treatment under other modalities. However, the study highlighted the need for anaesthetic provision for propofol delivery and supports previous literature that propofol TCI is not suited to operator-sedationist treatment as provided with midazolam due to the narrow margin of safety.

## Conclusion

This study adds to the current limited literature in reviewing IV sedation for adolescent patients requiring dental treatment. Several further areas of research have been identified including a qualitative investigation could explore the barriers to dental care for anxious adolescent patients and explore outcomes of patients and viewpoints following IV sedation. A RCT comparing patient safety and reported outcomes after sedation with IV midazolam compared with IV propofol for anxious adolescent patients would be interesting especially focusing on the recall and cognitive ability of patients during and after IV sedation.

Propofol can be used as a safe alternative sedation modality for the dental treatment of adolescent patients presenting with complex treatment needs and/or dental anxiety as it allows increased provision of treatment per visit with positive reported patient outcomes.
